# Relationship between serum brain-derived neurotrophic factor and cognitive impairment in children with sleep-disordered breathing

**DOI:** 10.3389/fped.2022.1027894

**Published:** 2023-01-05

**Authors:** Yani Feng, Lina Ma, Xi Chen, Yitong Zhang, Zine Cao, Yuqi Yuan, Yushan Xie, Haiqin Liu, Yewen Shi, Xiaoyong Ren

**Affiliations:** Department of Otorhinolaryngology Head and Neck Surgery, The Second Affiliated Hospital of Xi'an Jiaotong University, Xi’an, China

**Keywords:** sleep-disordered breathing, BDNF, trkB, cognitive impairment, pediatric

## Abstract

**Background:**

As an important neuroprotective factor, the brain-derived neurotrophic factor (BDNF) may have a key role in cognitive impairment in children with sleep-disordered breathing (SDB). The main aim of this study was to compare the levels of BDNF and tyrosine kinase receptor B (TrkB) in normal children and those with obstructive sleep apnea (OSA) and primary snoring (PS) and to explore a possible link between BDNF/TrkB, inflammation, and SDB with cognitive impairment in children.

**Methods:**

A total of 44 OSA children and 35 PS children who completed polysomnography between October 2017 and October 2019 were enrolled. At the same time, 40 healthy children during the same period were included as a control. Enzyme-linked immunosorbent assay was used to measure serum indices of BDNF, TrkB, interleukin-1beta (IL-1β), and tumor necrosis factor-alpha (TNF-α). Correlation and pooled analyses were performed between the cognitive scores and four serological indicators. Logistic regression was used to analyze the risk factors for cognitive impairment.

**Results:**

Significant differences were found in serum BDNF, TrkB, IL-1β, and TNF-α between the three groups (all *P* < 0.01). The serum BDNF and TrkB in the OSA and PS groups were lower than those in the control group, whereas the serum IL-1β and TNF-α were higher than those in the control group (all *P* < 0.05). Moreover, among these four indices, the strongest correlation was found between BDNF and the Chinese Wechsler Intelligence Scale (all *P* < 0.05). Logistic regression analysis revealed a correlation between OSA status, TrkB, and course of mouth breathing and cognitive status.

**Conclusion:**

The levels of serum BDNF and TrkB were related to cognitive impairment in children with SDB. Also, BDNF and TrkB could be used as noninvasive and objective candidate markers and predictive indices of cognitive impairment in children with SDB.

## Introduction

Sleep-disordered breathing (SDB) is a highly prevalent disease characterized by abnormalities in respiratory patterns, which cause arousals and affect the quantity of ventilation or arterial oxygen saturation (SaO_2_) during sleep, and it is characterized by intermittent hypoxia and sleep fragmentation. SDB is described as a spectrum of disorders from primary snoring (PS) to obstructive sleep apnea (OSA) on the basis of apnea indices from polysomnography (PSG) (1). According to the meta-analysis study of SDB in children, the prevalence of OSA ranged between 1% and 4%, and the prevalence of PS was as high as 7.45% (2). Nocturnal intermittent hypoxia and fragmented sleep caused by SDB can lead to adverse health consequences such as cognitive impairment (3). Regrettably, the research on the impact of SDB (including PS and OSA) on cognitive impairment in children was more focused on clinical research, with relatively few molecular mechanism studies compared with that on adults. Moreover, the assessment of cognitive impairment in children is usually performed using various scales, which may lead to subjective results. In this context, the exploration of objective and easily measurable biomarkers is of great importance in clinical practice.

Brain-derived neurotrophic factor (BDNF), a member of the neurotrophin family, is widely expressed in various brain regions. BDNF can pass through the blood–brain barrier, and its concentration in brain tissue and blood is closely related (4). Tyrosine kinase receptor B (TrkB) is a specific receptor for BDNF, and BDNF/TrkB signaling pathway has an important protective role in neuronal injury. Reduced BDNF levels in the human brain are associated with cognitive impairment caused by neurodegenerative diseases, such as Alzheimer's disease and Parkinson's schizophrenia (5, 6). In addition, Xie et al. used a mouse model of chronic intermittent hypoxia (IH) to provide convincing evidence for the key role of BDNF in OSA-induced cognitive impairment. They found that the expression of BDNF was significantly reduced after chronic IH in mouse. Also, supplementation of BDNF could rescue and prevent IH-induced long-term potentiation deficits (7, 8). SDB can induce a local and systemic inflammatory response, leading to the upregulation of inflammatory cytokines (9, 10), such as interleukin-1beta (IL-1β) and tumor necrosis factor-alpha (TNF-α), which could increase the permeability of the blood–brain barrier and lead to neuroinflammation or neurodegeneration with a consequent cognitive impairment (11–14). However, no clinical studies are confirming the relationship between BDNF and cognitive impairment in children with SDB, nor relevant research about detailed stratified analyses of PS and OSA in children.

It is now generally accepted that OSA is associated with cognitive and behavioral dysfunction in children (15, 16). Yet, the growing evidence showed that PS, the mildest and most common form of SDB, is not benign but carries a similar risk for cognitive and behavioral impairment as OSA in children (17). A review of the literature suggests, however, that the expression of serum BDNF and TrkB in PS children and their differences with OSA children have not been fully exploited. This study aimed to (1) investigate whether there are differences in the serum levels of BDNF and TrkB in OSA children, PS children, and healthy children, and (2) explore a possible link between BDNF/TrkB, inflammation, and SDB with cognitive impairment.

## Methods

### Study design and subjects

This cross-sectional observational study included 44 OSA children and 35 PS children diagnosed by PSG examination who were admitted to the Department of Otorhinolaryngology—Head and Neck Surgery of the Second Affiliated Hospital of Xi'an Jiaotong University between October 2017 and October 2019 because of habitual snoring during sleep. At the same time, 40 healthy children were recruited from the Children's Health Center of the same hospital as the control group. Inclusion and exclusion criteria are described in detail in the Supplementary Materials.

This study was approved by the ethics committee of the Second Affiliated Hospital of Xi'an Jiaotong University (approval no. 2017058). All children who participated in the research were accompanied by parents, who signed informed consent. The inspection items and processes involved in this study are in line with the declaration of Helsinki.

### Subject characteristics

All subjects underwent a routine medical history and physical examination by otolaryngologists, oral and maxillofacial surgeons, pediatricians, and child psychiatrists to assess comorbidities. In addition, gender, age, course of snoring, course of mouth breathing, course of chocking, paternal education level, maternal education level, body mass index (BMI), tonsil size, adenoidal/nasopharyngeal ratio, and Epworth Sleepiness Scale were recorded. Tonsil size was assessed using the Brodsky standardized system (18).

PSG is considered to be the gold standard for the diagnosis of SDB. All children who presented with habitual snoring underwent an overnight PSG assessment, during which relevant PSG data were collected, including apnea-hypopnea index, obstructive apnea index, obstructive apnea-hypopnea index (OAHI), rapid eye movement-respiratory disturbance index (REM-RDI), non-rapid eye movement-respiratory disturbance index (NREM-RDI), average SaO_2_, minimum SaO_2_, sleep efficiency, respiratory arousal index, REM respiratory arousal index, NREM respiratory arousal index. The obtained PSG data were scored according to the American Academy of Sleep Medicine guidelines (19). All subjects were grouped into PS and OSA groups according to OAHI. Also, OAHI was defined as the total number of obstructive apneas, mixed apneas, and obstructive hypopneas per hour of total sleep time. PSG data were evaluated and analyzed by two trained technicians, and were then reviewed by sleep specialists. Additionally, OSA diagnosis was based on OAHI ≥ 1 event per hour during PSG. PS was diagnosed in children with habitual snoring and OAHI < 1 event per hour (20).

### Measurement of serum indicators

The venous blood samples were drawn with a serum separator tube from all subjects in the morning between 6:30 AM and 7:00 AM and were centrifuged at 1,900 × g for 10 min to separate the upper serum, which was then stored at −80°C for subsequent use. Moreover, serum levels of BDNF (#RAB0026, Sigma-Aldrich), TrkB (#orb381119, Biorbyt), IL-1β (#QLB00B, R&D Systems), and TNF-α (#QTA00C, R&D Systems) were determined by enzyme-linked immunosorbent assay. Finally, the measurement of each sample was repeated three times, and the data were reported as the average of the three measurements. The inter-assay coefficient of BDNF, TrkB, IL-1β, and TNF-α was 3.6%, 4.0%, 3.4%, and 3.6%, respectively, and the intra-assay coefficient was 4.3%, 5.1%, 4.4%, and 4.1%, respectively.

### Behavioral and cognitive assessment

The Chinese Wechsler Intelligence Scale (C-WISC) and Conners' Parent Rating Scale (CPRS) were used to assess cognitive and behavioral function. The Wechsler Intelligence Sale (WISC) has three intelligence quotient (IQ) domains: verbal intelligence quotient (VIQ), performance intelligence quotient (PIQ), and full intelligence quotient (FIQ). C-WISC has been revised from the WISC for Children based on the Chinese cultural background, and FIQ < 90 means abnormal intelligence (21, 22). CPRS is a questionnaire designed for parents to assess their children's behavioral problems (23). Assessments were performed by two clinical psychologists who were blind to the subjects' clinical data.

### Statistical analysis

SPSS 21.0 (IBM Corporation, Armonk, NY, USA) and GraphPad Prism 7 (GraphPad Software Inc., San Diego, CA, USA) were used for data analysis. Descriptive characteristics across groups were compared using Chi square test or Fisher's exact test for categorical variables and nonparametric rank-sum test for grade data, and analysis of variance or for continuous variables. Continuous variables were tested for normality using the Shapiro–Wilk test, and the data with normal distribution were compared by analysis of variance or student's *t* test. Data with non-normal distribution were tested by nonparametric test. The relationships between continuous variables were explored using Pearson's correlation or Spearman's correlation.

A logistic regression model was used to assess the factors influencing the cognitive status. Logistic regression analysis was performed using intellectual status as the dependent variable (FIQ ≥ 90 indicates normal intelligence, FIQ < 90 indicates abnormal intelligence), and paternal education level, maternal education level, OSA status, course of snoring, course of choking, course of mouth breathing, Epworth Sleepiness Scale, gender, age, BMI, BMI-Z score, conduct problem, learning problem, somatic complaints, hyperactive, Conners' index of hyperactivity (CIH), anxiety, BDNF, TrkB, IL-1β and TNF-α as the independent variables were used for initially screening by using the least absolute shrinkage and selection operator of the independent variables. Further, the screened variables were included in logistic regression, adjusting for possible confounding factors such as age, sex, BMI-Z score. *P* < 0.05 was considered to be statistically significant.

## Results

### Subject characteristics

**Figure 1:**
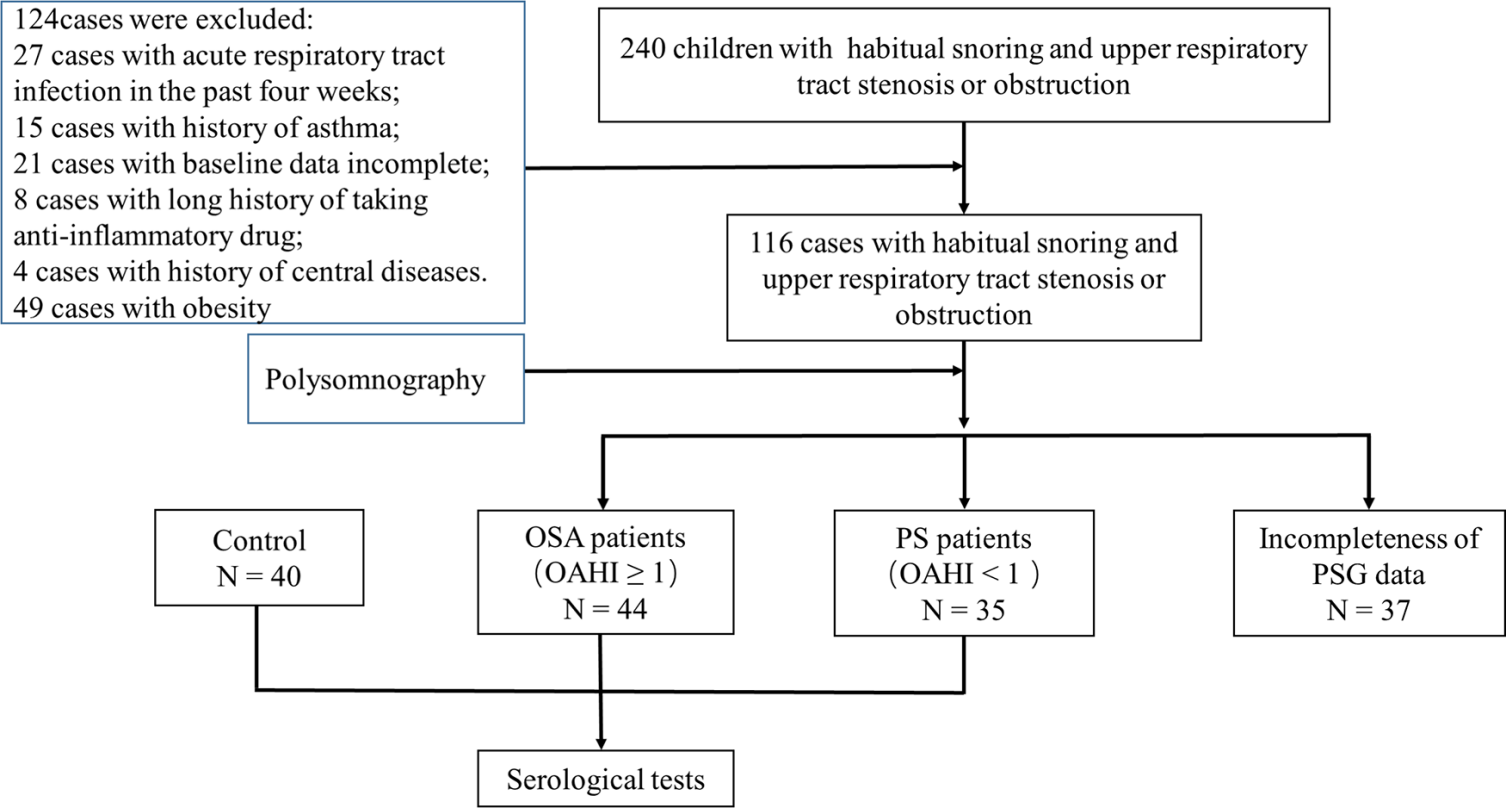
Summary of subject inclusion.

The patient flow in the current study is described in detail in Figure 1. After the inclusion and exclusion criteria were applied, this dataset included 119 children, with 44 children in the OSA group, 35 children in the PS group, and 40 healthy children in the control group.

The demographic and clinical characteristics of the groups are summarized in Table 1. There were no significant differences in age, BMI, BMI-Z score, and paternal education level among the three groups; however, there were statistically significant differences in gender (*P *< 0.05) and maternal education level (*P *< 0.05). In addition, there were no significant differences in other baseline data, including the course of snoring, course of mouth breathing, course of chocking, adenoidal/nasopharyngeal ratio, Epworth Sleepiness Scale, and tonsil size between the OSA group and the PS group.

**Table 1:** Comparisons of demographic and clinical characteristics among three groups.

	All	OSA patients	PS patients	control	*P*
Gender (male/female, *n*)^a^	119 (72/47)	44 (34/10)	35 (18/17)	40 (20/20)	<0.05
Age (year)^b^	6.97 ± 2.01	6.66 ± 1.96	7.43 ± 2.22	6.90 ± 1.83	0.234
Course of snoring (months)^c^	24.0 (8.0-36.0)	24.0 (12.0-36.0)	24.0 (5.0-36.0)	0.0 (0.0-0.0)	0.512
Course of mouth breathing (months)^c^	12.0 (5.0-24.0)	12.0 (5.3-24.0)	24.0 (5.0-36.0)	0.0 (0.0-0.0)	0.232
Course of choking (months)^c^	2.0 (0.0-12.0)	3.0 (0.0-12.0)	0.0 (0.0-6.0)	0.0 (0.0-0.0)	0.077
Epworth Sleepiness Scale^c^	4.0 (2.0-6.0)	4.0 (2.0-6.0)	4.0 (2.0-6.0)	N/A	0.568
BMI (kg/m^2^)^b^	16.44 ± 2.64	16.70 ± 2.95	16.37 ± 2.53	16.22 ± 2.39	0.699
BMI-Z score^b^	0.21 ± 1.26	0.30 ± 1.29	0.10 ± 1.07	0.21 ± 1.39	0.782
adenoidal/nasopharyngeal ratio^d^	0.68 ± 0.10	0.68 ± 0.10	0.67 ± 0.11	N/A	0.493
**Paternal education level^c^**					0.168
0	0	0	0	0	
1	5	1	2	2	
2	15	11	2	2	
3	20	8	6	6	
4	33	10	11	12	
5	40	12	12	16	
6	6	2	2	2	
**Maternal education level^c^**					<0.05
0	1	1	0	0	
1	4	0	2	2	
2	20	14	4	2	
3	20	9	5	6	
4	25	8	7	10	
5	44	11	15	18	
6	5	1	2	2	
**Tonsil size (*n*)^c^**					0.060
0	3	0	3	N/A	
1	3	2	1	N/A	
2	31	14	17	N/A	
3	6	5	1	N/A	
4	36	23	13	N/A	

BMI, body mass index; The education level: 0, without education; 1, completed primary school; 2, completed part secondary education; 3, completed secondary education; 4, completed postsecondary training; 5, completed an undergraduate university degree; 6, completed a postgraduate university degree; OSA,: obstructive sleep apnea; PS, primary snoring.

^a^ Chi-squared test or Fisher's exact test.

^b^ Analysis of variance test.

^c^ Nonparametric test.

^d^ The student's *t* test.

The PSG results of OSA group and PS group are shown in Table 2. As expected, most of PSG parameters were significantly different between the two groups (*P *< 0.0001), except for sleep efficiency.

**Table 2:** Comparisons of PSG characteristics of the OSA and PS patients.

	All	OSA patients	PS patients	*P*
AHI (events/h)	6.14(0.70–12.00)	8.99(6.78-18.54)	0.70(0.30–0.90)	<0.0001
OAI (events/h)	0.69 (0.10–2.50)	2.20 (1.30–4.00)	0.10 (0.00–0.30)	<0.0001
OAHI (events/h)	2.90(0.30–7.40)	6.81(4.49–14.67)	0.20(0.10–0.40)	<0.0001
REM-RDI (events/h)	7.20 (1.10–14.07)	13.19 (8.95–30.63)	0.70 (0.50–1.89)	<0.0001
NREM-RDI (events/h)	5.09(0.70–10.29)	8.54 (5.41–15.41)	0.65(0.20–0.89)	<0.0001
Average SaO_2_ (%)	97.00 (96.00–98.00)	97.00 (96.00–97.00)	98.00 (97.00–98.00)	<0.0001
Minimum SaO_2_ (%)	89.00(85.00–92.00)	86.00(81.00–89.00)	92.00(90.00–94.00)	<0.0001
Sleep efficiency (%)	93.30 (85.60–96.20)	93.20 (86.78–96.13)	93.80 (85.50–96.30)	0.813
Respiratory arousal index	0.60 (0.00–2.10)	1.75 (1.00–3.98)	0.00 (0.00–0.10)	<0.0001
REM respiratory arousal index	0.60 (0.00–3.30)	2.60 (0.73–5.20)	0.00 (0.00–0.00)	<0.0001
NREM respiratory arousal index	0.50 (0.00–1.90)	1.35 (0.63–3.48)	0.00 (0.00–0.10)	<0.0001

PSG, polysomnography; AHI, apnea-hypopnea index; OAI, obstructive apnea index; OAHI, obstructive apnea-hypopnea index; REM–RDI, rapid eye movement–respiratory disturbance index; NREM–RDI, non–rapid eye movement–respiratory disturbance index; SaO_2_, arterial oxygen saturation; OSA, obstructive sleep apnea; PS, primary snoring. Nonparametric test has been applied to investigate for between group differences.

### Serum indicators, behavioral and cognitive assessment

The differences in cognitive and behavioral scores between groups are shown in Table 3. In the CPRS, a comparison among the three types of children showed significant differences in learning problems and hyperactivity; however, there was no significant difference in the other four indices in the three groups. Additionally, compared with the patients in the PS group, the patients in the OSA group had significantly lower FIQ, VIQ, and PIQ (all *P* < 0.05).

**Table 3:** Comparisons of cognitive and behavioral function tests between groups.

	All	OSA patients	PS patients	Control	*P*
Conduct problem^a^	0.45 (0.07–0.65)	0.38 (0.00–0.90)	0.45 (0.17–0.55)	0.42 (0.17–0.58)	0.773
Learning problem^a^	1.00 (0.50–1.25)	1.00 (0.53–1.25)	0.85 (0.50–1.25)	0.75 (0.25–1.00)	<0.05
Somatic complaints^a^	0.20 (0.00–0.40)	0.20 (0.00–0.40)	0.00 (0.00–0.20)	0.00 (0.00–0.40)	0.077
Hyperactivity^a^	0.75 (0.50–1.25)	0.75 (0.50–1.25)	0.75 (0.50–1.25)	0.50 (0.25–1.00)	<0.05
CIH^a^	0.40 (0.20–0.80)	0.45 (0.33–0.80)	0.40 (0.20–0.90)	0.50 (0.20–0.95)	0.786
Anxiety^a^	0.30 (0.00–0.50)	0.40 (0.25–0.50)	0.25 (0.00–0.75)	0.25 (0.00–0.50)	0.921
PIQ^b^	94.70 ± 13.76	91.07 ± 12.27	99.26 ± 14.34	N/A	<0.01
VIQ^b^	96.78 ± 13.48	93.55 ± 12,42	100.86 ± 13.82	N/A	<0.05
FIQ^b^	93.47 ± 14.56	89.91 ± 12.38	97.94 ± 15.99	N/A	<0.05

CIH, Conners’ index of hyperactivity; PIQ, Performance intelligence quotient; VIQ, Verbal intelligence quotient; FIQ, Full intelligence quotient; OSA, Obstructive sleep apnea; PS, Primary snoring.

^a^ Nonparametric test.

^b^ The student's *t* test.

**Figure 2:**
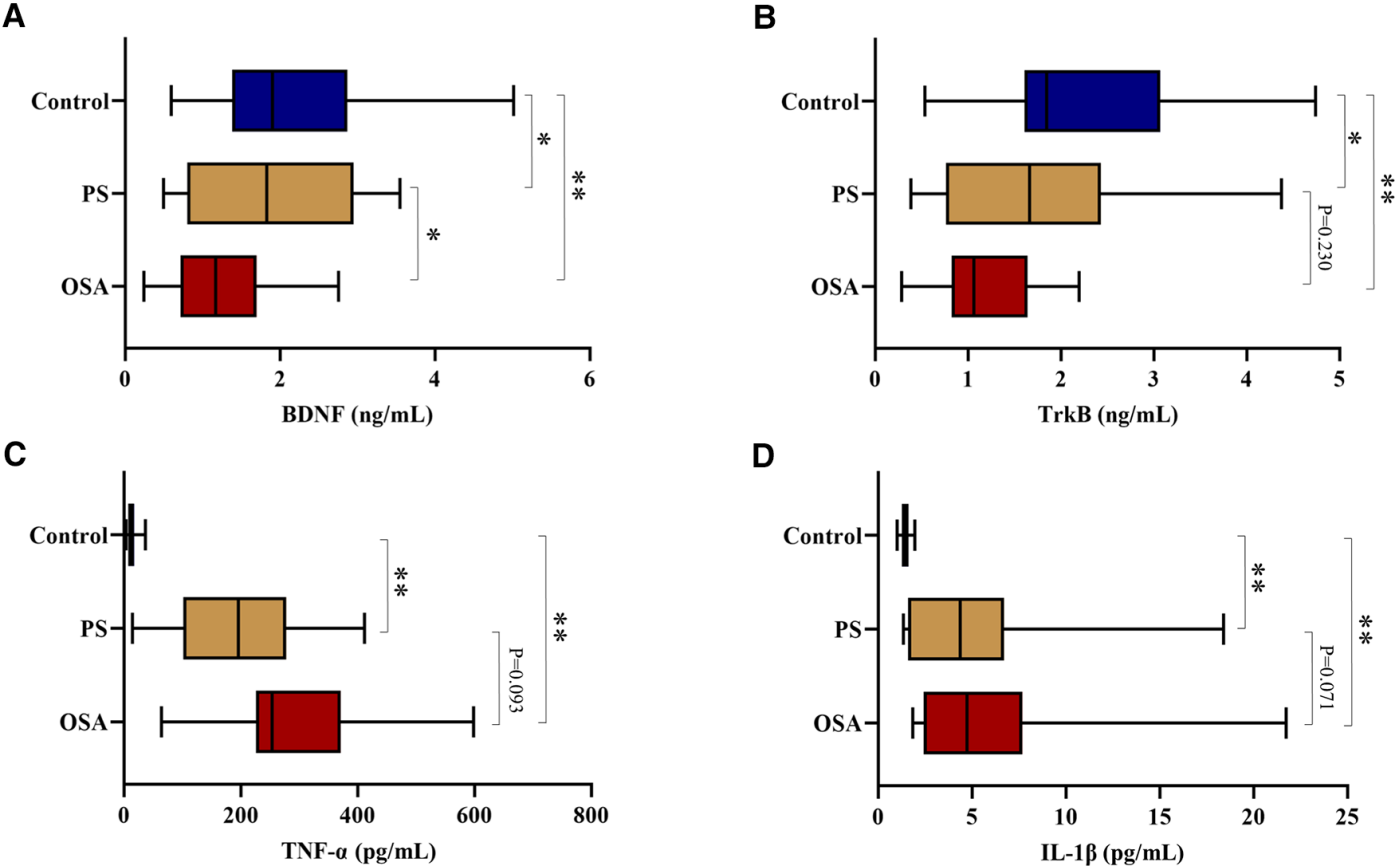
(**A**) Comparison of serum BDNF levels among three groups; (**B**) Comparison of serum TrkB levels among three groups; (**C**) Comparison of serum TNF-α levels among three groups; (**D**) Comparison of serum IL-1β levels among three groups. **P < 0.01, *P < 0.05. BDNF, brain-derived neurotrophic factor; TrkB, tyrosine kinase receptor B; IL-1β, interleukin-1beta; TNF-α, tumor necrosis factor-alpha.

The serum levels of BDNF and TrkB in OSA and PS children were significantly lower than those of the control group (all *P* < 0.05), especially in the OSA group. Furthermore, there were also significant differences in IL-1β and TNF-α among the three groups (*P* < 0.001), with a significant increase in the OSA and PS groups (Table 4; Figure 2).

**Table 4:** Comparisons of BDNF, trkB, IL-1β and TNF-α among three groups.

Variables	OSA patients	PS patients	Control	*P*
BDNF (ng/ml)	1.22 ± 0.63	1.78 ± 1.01	2.35 ± 1.30	<0.001
TrkB (ng/ml)	1.21 ± 0.55	1.62 ± 0.99	2.24 ± 1.07	<0.001
TNF-α (pg/ml)	284.59 ± 122.76	194.88 ± 121.21	13.89 ± 8.74	<0.001
IL-1β (pg/ml)	6.80 ± 5.65	4.61 ± 3.86	1.45 ± 0.26	<0.001

BDNF, Brain–derived neurotrophic factor; TrkB, Tyrosine kinase receptor B; IL–1β, interleukin–1beta; TNF–α, tumor necrosis factor–alpha; OSA, obstructive sleep apnea; PS, primary snoring. Analysis of variance test has been applied to investigate for between group differences.

### Correlation analysis

Correlation analysis showed a good correlation between BDNF and TrkB (*r* = 0.919, *P *< 0.01), IL-1β (*r* = −0.312, *P *< 0.01) and TNF-α (*r* = −0.507, *P *< 0.01). Besides, there was also a correlation between BDNF and OAHI (*r* = −0.315, *P *< 0.01), and it was worth noting that there was a correlation between BDNF and NREM−RDI rather than REM−RDI (*r* = −0.356, *P *< 0.01). In CPRS, only hyperactivity was correlated with BDNF (*r* = −0.241, *P *< 0.01). In C-WISC, the Pearson correlation, which was carried out for VIQ, PIQ, FIQ, and BDNF, revealed a positive correlation (VIQ: *r* = 0.317, *P *< 0.01; PIQ: *r* = 0.300, *P* < 0.01; FIQ: *r* = 0.249, *P *< 0.05). Furthermore, the positive correlation was also observed between TrkB and FIQ (*r* = 0.316, *P *< 0.01), and PIQ (*r* = 0.307, *P *< 0.01) (Table 5).

**Table 5:** Correlation analysis between baseline characteristics, inflammatory indicators, behavioral and cognitive parameters.

Variables	BNDF		TrkB		TNF-α		IL-1β	
	Correlation coefficient	*P*	correlation coefficient	*P*	correlation coefficient	*P*	correlation coefficient	*P*
BDNF	1.000	-	0.919	<0.01	−0.507	<0.001	−0.312	<0.01
TrkB	0.919	<0.01	1.000	-	−0.570	<0.001	−0.350	<0.01
TNF-α	−0.507	<0.01	−0.570	<0.01	1.000	-	0.741	<0.01
IL-1β	−0.312	<0.01	−0.350	<0.01	0.741	<0.001	1.000	-
Course of snoring	−0.147	0.110	−0.214	<0.05	0.588	<0.001	0.498	<0.01
Course of mouth breathing	−0.194	<0.05	−0.240	<0.01	0.590	<0.001	0.575	<0.01
Course of choking	−0.170	0.065	−0.232	<0.05	0.446	<0.001	0.419	<0.01
Epworth sleepiness scale	−0.065	0.483	−0.067	0.467	0.050	0.593	0.120	0.192
BMI	−0.073	0.429	−0.071	0.441	−0.017	0.857	−0.340	0.711
BMI-Z score	−0.033	0.718	−0.50	0.592	−0.034	0.711	−0.095	0.302
A/N ratio	−0.204	0.071	−0.186	0.101	−0.038	0.740	−0.007	0.948
Tonsil size	−0.242	<0.05	−0.199	0.079	0.075	0.511	0.014	0.905
Maternal education level	0.135	0.143	0.079	0.393	−0.055	0.553	−0.153	0.097
OAHI	−0.315	<0.01	−0.224	<0.05	0.261	<0.05	0.264	<0.05
REM-RDI	−0.195	0.084	−0.129	0.257	0.278	<0.05	0.246	<0.05
NREM-RDI	−0.356	<0.01	−0.261	<0.05	0.323	<0.01	0.302	<0.01
Conduct problem	−0.090	0.330	−0.062	0.505	−0.009	0.920	−0.044	0.636
Learning problem	0.002	0.984	0.052	0.575	0.199	<0.05	0.184	<0.05
Somatic complaints	−0.091	0.327	−0.074	0.424	0.180	0.050	0.233	<0.05
hyperactivity	−0.241	<0.01	−0.206	<0.05	0.272	<0.01	0.210	<0.05
Anxiety	0.187	<0.05	0.151	0.101	−0.082	0.377	0.058	0.532
CIH	−0.029	0.754	0.008	0.119	−0.051	0.585	−0.012	0.895
FIQ	0.249	<0.05	0.316	<0.01	−0.218	0.054	−0.117	0.304
VIQ	0.317	<0.01	0.220	0.051	−0.246	<0.05	−0.135	0.237
PIQ	0.300	<0.01	0.307	<0.01	−0.272	<0.05	−0.146	0.199

BDNF, brain–derived neurotrophic factor; TrkB, tyrosine kinase receptor B; IL-1β, interleukin-1beta; TNF-α, tumor necrosis factor-alpha; BMI, body mass index; OAHI, obstructive apnea-hypopnea index; REM-RDI, rapid eye movement-respiratory disturbance index; NREM-RDI, non-rapid eye movement-respiratory disturbance index; CIH, Conners’ index of hyperactivity; FIQ, full intelligence quotient; VIQ, verbal intelligence quotient; PIQ, performance intelligence quotient.

**Figure 3:**
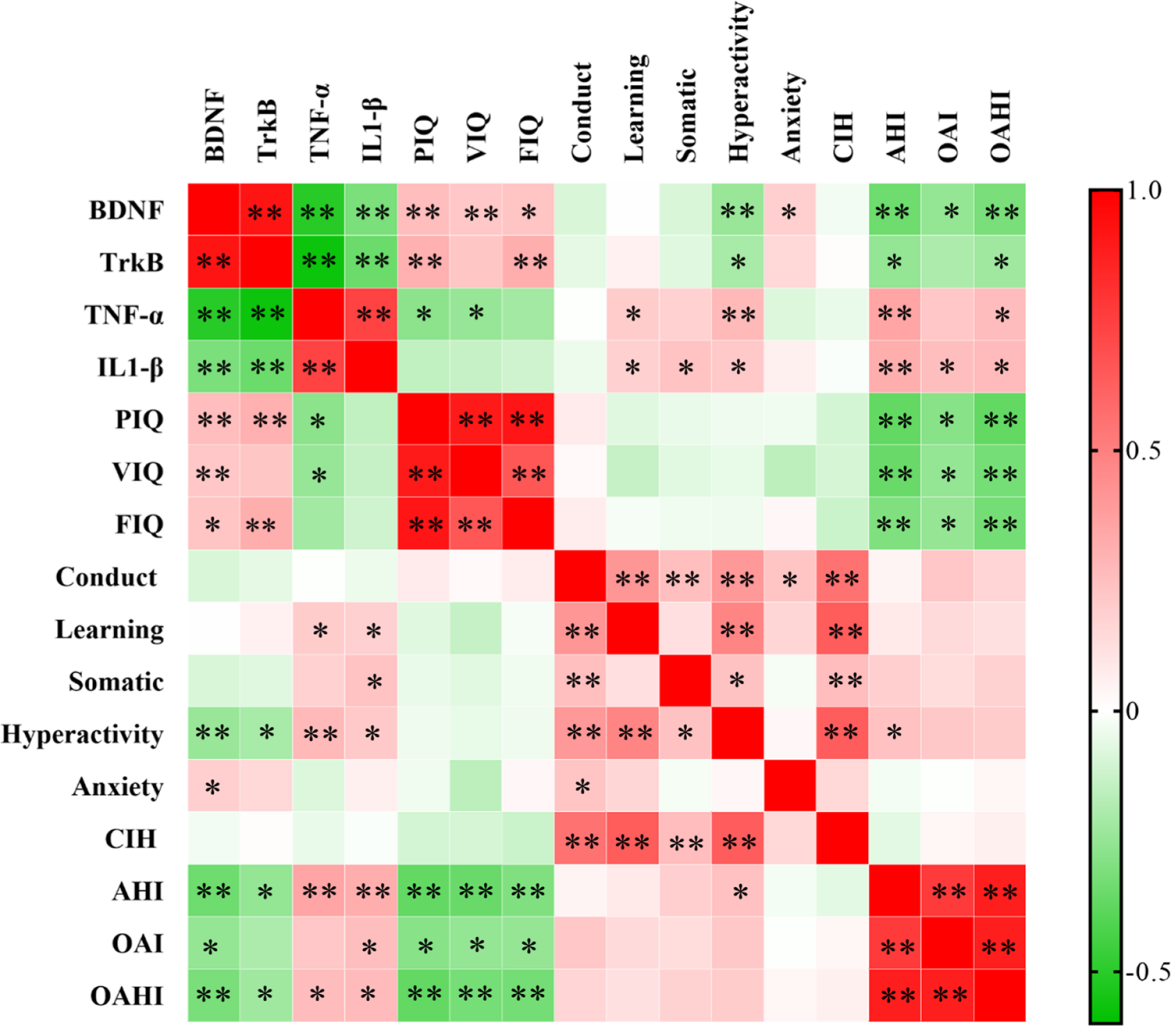
Correlation analysis between BDNF, TrkB, IL-1β, TNF-α, and cognitive parameters. **P < 0.01, *P < 0.05. BDNF, brain-derived neurotrophic factor; TrkB, tyrosine kinase receptor B; IL-1β, interleukin-1beta; TNF-α, tumor necrosis factor-alpha.

Additionally, a negative correlation was found between TNF-α and VIQ (*r* = −0.246, *P *< 0.05) and PIQ (*r* = −0.272, *P *< 0.05), whereas no correlation was found between IL-1β and PIQ, VIQ, or FIQ (Figure 3).

### Analysis of factors influencing the cognitive status in subjects

As evident from Table 6. The results showed the association of OSA status, course of mouth breathing and TrkB with intellectual status. Specifically, in analyses adjusted for age, sex and BMI-Z score, OSA children had a higher risk of cognitive impairment than those with an OAHI < 1 [odds ratio (OR) = 4.582, 95% confidence interval (CI): 1.496 to 14.034], and beneficiaries who with high levels of TrkB had a significantly lower odds of developing cognitive impairment (OR = 0.362, 95% CI: 0.157 to 0.836). Additionally, the longer duration of mouth breathing also increases the risk of cognitive impairment (OR = 1.043, 95% CI: 1.010 to 1.077).

**Table 6:** Results of the logistic regression analysis: assessing the risk of abnormal intelligence in relation to different independent variables.

Variables	Unadjusted odds ratio (95% CI)	*P*	Adjusted odds ratio (95% CI)	*P*
**OSA status**				
OSA	4.802 (1.678–13.744)	<0.01	4.582 (1.496–14.034)	<0.01
Non-OSA	Ref	Ref	Ref	Ref
Couse of mouse breathing	1.045 (1.014–1.077)	<0.01	1.043 (1.010–1.077)	0.01
TrkB	0.373 (0.166–0.839)	<0.05	0.362 (0.157–0.836)	<0.05

TrkB, tyrosine kinase receptor B; OSA, obstructive sleep apnea; CI, confidence interval; Adjusted models controlled for age, sex and BMI-Z score.

## Discussion

In this study, we investigated the relationship between cognitive status and serum BDNF and TrkB in OSA and PS children. In addition, we discovered that the serum BDNF level in children with SDB was lower than that of the control group, especially in the OSA group. Furthermore, we revealed a correlation between OSA status, TrkB, and course of mouth breathing and cognitive status through logistic regression analysis.

In the present study, we found that the maternal education level in the OSA group was significantly lower compared to the control group. This may suggest that mothers with high educational levels were more conducive to their children's early medical treatment. Interestingly, no significant difference was found between BMI and BMI-Z scores between the groups, which was not consistent with a previous study (2). This may be caused by the outdated belief that heavier children were closely associated with snoring, thus resulting in a higher diagnosis among heavier children. However, this still needs to be verified by larger sample sizes in the future.

Over the past several decades, numerous studies have shown that children who suffer from SDB are at great risk for behavioral disorders, which may manifest in a variety of ways. The most commonly observed disorders are hyperactivity disorder, attention disorder, and somatic complaints (17, 24). According to the results of CPRS among the three groups, the OSA children were found to have behavioral disorders, mainly learning problems and hyperactivity, whereas the PS children did not show any obvious behavioral disorders when compared with the control group. We observed that the average scores of the PS children were much higher than those of the control group. We speculated that this might be because PS children generally have a shorter course of the disease and inconspicuous symptoms than OSA children. Thus, PS children received less attention from parents, which in turn affected parents' subjective tendencies when filling out the scale. Consequently, further validation by large sample studies is required.

We used C-WISC combined with BNDF and TrkB to explore the cognitive impairment in children with SDB. The comparison of intelligence between the two groups showed that VIQ, PIQ, and FIQ of OSA children were significantly lower than those of PS children, indicating that the cognitive functions were worse in children from the OSA group. Regrettably, we were unable to assess the level of cognitive impairment in PS children because of the lack of C-WISC scores for children in the control group. Nevertheless, compared with the C-WISC scores of normal children in previous studies (25), the scores of PIQ, VIQ, and FIQ in the PS group were not significantly lower. However, this did not suggest that cognitive impairment does not exist in PS children. As our results revealed a significant increase in inflammatory factors and a decrease in BDNF and TrkB in PS children like OSA children, this suggested that PS children experienced similar inflammatory responses and nerve injury as OSA children. Nonetheless, we did not find weak hypoxia or arousal in PS children, which may be caused by the lack of precision and interpretation accuracy of PSG. It is also possible that hypoxia or arousal in PS children did not happen during PSG detection. It is necessary to acknowledge that even though PSG suggests that PS children do not have nocturnal hypoxia, this does not mean that they do not have cerebral-level hypoxia.

A few studies have looked at the relationship between BDNF and OSA in adults and showed conflicting results, as some have found a relationship between BDNF and OSA (26, 27), whereas others reported no significant difference (28, 29). In addition, the association between circulating levels of BDNF and children with SDB is also still unclear. Consistent with our findings, Bahgat et al. have previously shown that OSA children have lower serum BDNF levels than healthy controls (30). Moreover, a significant difference was also found in serum BDNF levels between OSA children and PS children. In contrast, studies by Makhout et al. and Wang et al. have shown that BDNF levels are not affected by OSA in children (31, 32). The results and patients were not evaluated in terms of OAHI ≥ 1 in these studies, which could explain the discrepancy in the results. However, these studies failed to explore the relationship between BDNF and PS children and the relationship between BDNF and cognition. At the same time, correlation analysis showed a good correlation between the level of serum BNDF and the cognitive scores in our study, indicating that the higher the level of serum BDNF, the better the score of cognitive assessment, and suggesting that BDNF may reflect the severity of cognitive impairment in children with SDB. Interestingly, children with lower serum TrkB levels, but not serum BDNF, were at a higher risk of abnormal intelligence. This suggests that TrkB, a receptor for multiple neurotrophins (BDNF and neurotrophin 4) (33), may be a more sensitive and worthy indicator of cognitive impairment than serum BDNF. In addition, serum TrkB is a marker that is easier to detect than BDNF, which is unstable in blood (34).

As a harmful consequence induced by chronic IH, apoptotic neuronal cell death is strongly believed to contribute to cognitive impairment in OSA disease, as shown in animal models and clinical studies (35, 36). And may be a major medium. As previously mentioned, chronic IH associated with SDB can lead to the release of pro-inflammatory cytokines that activate apoptotic signals and damage the hippocampus and neurons (10), which may contribute to the decline in BDNF and TrkB expression. The decline of BDNF and TrkB further aggravates neurological damage and eventually leads to cognitive impairment (7). The present study also confirmed the aforementioned results, as we found that the inflammatory factors TNF-α and IL-1β were significantly increased in children with SDB and that TNF-α was negatively correlated with VIQ and PIQ, thus suggesting that the inflammatory response in children with SDB may affect their cognitive status. Moreover, both BDNF and TrkB were negatively correlated with TNF-α and IL-1β, which may indicate that the inflammatory response has a certain inhibitory effect on the expression of BDNF and TrkB. This also explained why there were significant differences in BDNF rather than IL-1β and TNF-α between PS and OSA groups, as inflammatory markers appeared relatively earlier.

To the best of our knowledge, the current study is the first to assess the relationship between cognitive status and serum BDNF, TrkB in children with OSA and PS. Nonetheless, present study still has some limitations. First, future large-scale, multicenter, hospital-community prospective studies are needed to assess whether BDNF can reflect the degree of cognitive impairment and whether it can be used as a reliable predictor of cognitive impairment in children with SDB. Second, in our study, we included only younger children (3–12 years), therefore, our results cannot be interpolated to older children and adolescents. Third, in the control group, we excluded children with SDB by using the medical history, Pediatric Sleep Questionnaire, and physical examination, and we did not perform PSG and C-WISC.

## Conclusion

Our findings showed that serum BDNF and TrkB in OSA and PS children were lower than those in the control group and that serum BDNF was positively correlated with PIQ, FIQ, and VIQ. Serum BDNF and TrkB levels may be promising biological indicators reflecting the severity of cognitive impairment and predicting cognitive impairment in children with SDB.

## Data Availability

The raw data supporting the conclusions of this article will be made available by the authors, without undue reservation.
